# Is Individualism Suicidogenic? Findings From a Multinational Study of Young Adults From 12 Countries

**DOI:** 10.3389/fpsyt.2020.00259

**Published:** 2020-04-03

**Authors:** Mehmet Eskin, Ulrich S. Tran, Mauro Giovanni Carta, Senel Poyrazli, Chris Flood, Anwar Mechri, Amira Shaheen, Mohsen Janghorbani, Yousef Khader, Kouichi Yoshimasu, Jian-Min Sun, Omar Kujan, Jamila Abuidhail, Khouala Aidoudi, Seifollah Bakhshi, Hacer Harlak, Maria Francesca Moro, Louise Phillips, Motasem Hamdan, Abdulwahab Abuderman, Kanami Tsuno, Martin Voracek

**Affiliations:** ^1^Department of Psychology, College of Social Sciences and Humanities, Koç University, Istanbul, Turkey; ^2^Department of Cognition, Emotion, and Methods in Psychology, School of Psychology, University of Vienna, Vienna, Austria; ^3^Wiener Werkstaette for Suicide Research, Vienna, Austria; ^4^Department of Public Health, Clinical and Molecular Medicine, University of Cagliari, Cagliari, Italy; ^5^School of Behavioral Sciences and Education, Pennsylvania State University-Harrisburg, Middletown, PA, United States; ^6^School of Health Sciences, City, University of London, London, United Kingdom; ^7^Department of Psychiatry, University Hospital of Monastir, Monastir, Tunisia; ^8^Faculty of Medicine and Health Sciences, An-Najah National University, Nablus, West Bank, Palestine; ^9^School of Public Health, Isfahan University of Medical Sciences, Isfahan, Iran; ^10^Department of Community Medicine, Public Health and Family Medicine, Faculty of Medicine, Jordan University of Science & Technology, Irbid, Jordan; ^11^Department of Hygiene, School of Medicine, Wakayama Medical University, Wakayama, Japan; ^12^Department of Management and International Business, Faculty of Business and Economics, University of Auckland, Auckland, New Zealand; ^13^UWA Dental School, The University of Western Australia, Perth, WA, Australia; ^14^Faculty of Nursing, The Hashemite University, Zarqa, Jordan; ^15^Department of Psychology, Faculty of Arts and Sciences, Adnan Menderes University, Aydin, Turkey; ^16^School of Public Health, Al-Quds University, Jerusalem, Palestine; ^17^College of Medicine, Prince Sattam Bin Abdulaziz University, Al-Kharj, Saudi Arabia; ^18^School of Health Innovation, Kanagawa University of Human Services, Kawasaki, Japan

**Keywords:** suicidal behaviour, attitudes, psychological distress, individualism, collectivism, multination study

## Abstract

The associations of individualistic versus collectivistic value orientations with suicidal ideation and attempts, attitudes towards suicide and towards suicidal individuals, and psychological distress were investigated across 12 nations (*N* = 5572 university students). We expected differential associations of value orientations with suicidal behavior and moderating effects of the prevailing value orientations in the various countries. Findings showed that intermediate levels of individualism appeared protective against suicide attempts across all investigated nations, but that, otherwise, there seemingly are no universal associations of individualism and collectivism with suicidal behaviors. High collectivism was associated with less suicidal ideation only in individualistic countries. Low individualism appeared to be a risk factor for suicidal ideation specifically in Muslim collectivistic cultures, whereas high individualism in Asian collectivistic cultures. Collectivistic values are uniformly associated with less permissive attitudes to suicide, whereas individualistic values with a more stigmatized view of suicidal behavior. Both individualistic and collectivistic values were associated with socially accepting attitudes to a suicidal peer, helping a suicidal friend, and emotional involvement. The associations of individualistic and collectivistic values with disapproving attitudes to suicidal disclosure were complex. Beliefs in punishment after death for suicide, seeing suicide as mental illness, and emotional involvement with a suicidal friend were lower in high-suicide-rate countries. These evidence patterns are discussed in the light of related research evidence, along with directions for future research in this area.

## Introduction

Suicidal behavior shows both interpersonal and intersocietal variations (1, 2). The cultural contents such as values, codes, and attitudes with regards to suicide oftentimes are seen as contributing to such variations. For instance, research suggests that cultural approval of or permissive cultural attitudes towards suicide are associated with increased propensity toward suicide (3–5). Culture is a self-evident and ubiquitous, yet elusive, concept. To achieve scientific precision and progress, there is a need to unpack the contents and components of this all-inclusive concept. What exactly is meant and implied by culture? Are the contents, or ingredients of culture associated with an increased or decreased propensity for suicidal behavior and psychological distress?

Cultural or cross-cultural psychology has identified individualism-collectivism as a meaningful dimension, along which cultures and/or cultural groups can be compared and contrasted (6, 7). Qualities such as personal autonomy, self-reliance, uniqueness, and independence are valued in individualistic cultures but person-other relatedness or interdependence, and the person as being a part of a collective are the qualities that are valued in collectivistic cultures (8). Individualistic and collectivistic values (9) influence psychological variables, such as self-concept, motivation, affect, cognition, cognitive processing style, attribution, emotion regulation, and social support provisions (10–15).

The debate on the influence of individualism and collectivism on suicidal behavior is not new in suicidology. For instance, Durkheim (16) saw the causes of suicidal behavior in the relationship between the person and the collective. Hence, individualism and collectivism are about the relationship between the individual and the collective. Values such as self-reliance, personal uniqueness, independence, and those setting priority on personal goals are regarded central in individualistic cultures, but interdependence, relatedness, and values setting priority on group goals (8) are seen central in collectivistic cultures. Although the cultural dimension of individualism-collectivism provides an important source of information about intersocietal and interindividual differences, it nevertheless has not yet been fully integrated into suicide research.

As societies differ on individualism-collectivism dimension, they also differ on the prevalence of suicidal behaviors. For instance, Hansen and Pritchard (17) showed that suicide rates in 22 developed countries presented consistent patterns over time. Like most human behavior, culture exerts an influence on suicide (18, 19). For a better understanding of suicidal phenomena, some suicidologists therefore have called for an inclusion of culture in suicide research (20, 21). Such an inclusion of culture in suicide research raises three questions. The first one is: What do we mean by culture, and how do we conceptualize it, in order to include it in suicide research? This relates to the issue of precision. The second question is: How does culture affect suicide? This relates to the issue of mechanisms or processes. The third one is: Do we conceptualize culture at the group level or individual level? This relates to the level of measurement.

Eskin (22) has argued that individualistic and collectivistic values might influence the onset, maintenance, and aggravation of suicidal feelings in two important ways. In the first, during times of crises persons with individualistic values may take responsibility for what happened and thus may blame themselves. This may further aggravate the predominant feelings in a suicidal process such as anger, unhappiness, and hopelessness. On the other hand, in a similar situation, individuals with collectivistic values may attribute responsibility to others or to situations, which in turn may diminish the impact of these feelings.

Second, individualism and collectivism may exert influences on attitudes to suicide and attitudes to suicidal persons. The defining features of individualism include independence, freedom, choice, personal responsibility, and competition (23, 24). The act of suicide frequently is depicted as involving personal freedom and choice (25, 26). In line with this, suicidal individuals usually do not seek help (27, 28). Scientific studies indicate that perceived stigma, embarrassment, and a preference for self-reliance are the most common barriers to help-seeking (29–31). Within a psychological value matrix like this one, it is reasonable to assume that people with an individualistic worldview will see suicide as an act that is compatible with a worldview that stresses personal freedom and choice. Subsequently, they might show higher acceptance levels of suicide than those with a collectivistic worldview. Persons with an individualistic value orientation have a context-independent information processing style (12), and hence they may see the causes for a suicidal act as situated within the person and hence blame the person for what happened. On the contrary, persons with a collectivistic value orientation have a context-dependent information processing style (12), with a collectivistic mind set they may see the agent of a suicidal act as a victim and blame the situation or others for what happened. Extant evidence from cross-national comparative research suggests that suicide attempters who live in individualistic cultures may not receive the help they need. In contrast, suicide attempters who live in collectivistic cultures may receive the help they need (32–34). However, opposing findings have also been reported in a comparative investigation of Australia, India, and Italy (35).

The interaction between culture and person variables is seen as a determinant of suicidal behavior. Individualistic and collectivistic values relate to the relationship between the person and the collective. Being situated at a certain point on this dimension may involve advantages or disadvantages for both the collective and the person. Researchers seem to hold conflicting views about the benefits and harms of being situated on a certain point on the individualism-collectivism dimension with regards to mental health and well-being. For instance, Eckersley (36) opines that materialism and individualism are health hazards for population health. On the other hand, for Veenhoven (37) individualism fits human nature better than collectivism. If indeed this is the case, then individualism should promote better social and personal mental well-being. It is informative to review what empirical evidence is available on this point.

Research suggests that, for both societies and individuals, having individualistic values is associated with increased rates of completed suicide and suicidal behavior. Ecologic (group-level, or geographic) studies yield positive associations between individualism and suicide (4, 38–40). Evidence from individual-level investigations research confirms these group-level effects. For example, Leeuwen et al. (41) found individualistic values to be risk factor for suicidal ideation among immigrant adolescents in France. In a study of Australian university students Scott et al. (42) found students with strong individualistic values (idiocentrism) to be less satisfied with, and less inclined to seek social support, and presenting higher levels of hopelessness and suicide ideation. In Turkish adolescents and young adults, Eskin (22) showed suicidal thoughts and attempts to be more common among participants with individualistic than among those with collectivistic tendencies. The same study further showed that, although participants with individualistic tendencies held more permissive attitudes to suicide, they were less accepting of a suicidal close friend than those with collectivistic tendencies. In yet another study with Chinese participants, Du et al. (43) similarly showed individualistic orientation to be associated with increased hopelessness and substance use, along with reverse associations for collectivistic orientation.

Other lines of research, however, are suggestive for beneficial effects of individualism on psychological well-being. In these investigations, it is assumed that individualism exerts a positive effect on mental well-being *via* its potentials for creating a context for freedom and choice. Some aggregate-level data suggest positive associations of individualistic values with happiness and psychological well-being (37, 44, 45). However, one should be aware of the fact that these findings mostly stem from ecologic studies. From a methodological point of view, ecologic research designs are prone to confounders (cross-level bias) and need to be confirmed by individual-level evidence.

Yet another line of inquiry has argued that what matters for a better mental health is the person-environment fit (46). This line of reasoning assumes that persons at the extreme ends of the individualism-collectivism dimension, which, when incompatible with the societal values, have disadvantages for personal adaptation. The data seem to support this view. For instance, evidence (47) from Turkish and US students residing in the respective countries showed that having a value orientation inconsistent with societal values was associated with poor mental health. An investigation of Japanese and US students residing in the respective countries (48) yielded evidence that individualistic values were negatively correlated with the number of close friends and with subjective well-being among Japanese students, but not among US students.

There are some clear gender differences in suicidal behavior. In general, women contemplate and attempt suicide more often than men, but more men than women kill themselves (49). This is known as “gender paradox” in suicidal behavior (50, 51) and relates to the gender culture. The paradox has usually been explained through reference to the choice of method for and intent involved in suicidal behavior. The scientific investigations provide support for the view that men make use of more lethal methods for their suicidal behavior than women (52) but women and men are found to be similar in their intent lethality (53). The choice of more lethal methods for suicidal behavior by men is in line with the cultural gender stereotypes (54).

There is considerable scholarly debate on the conceptualization and measurement of individualism and collectivism constructs (55). Although most researchers view individualism and collectivism as opposites of a dimension, others see it as two separate orthogonal constructs (7, 56). Utilizing confirmatory factor analysis, Li and Aksoy (57) showed that individualism and collectivism represent two different constructs. Although we have introduced individualism and collectivism as one dimension for ease of comprehension, we use them as two orthogonal constructs in analysis in this multinational study. This approach may better enable us to see the individual effects of the two value orientations on suicidal behavior, suicidal attitudes, and psychological distress.

To sum up, the research literature suggests that individualistic and collectivistic values may have advantages and disadvantages for psychological well-being. Empirical evidence for possible relations of individualism-collectivism to suicidal behavior and psychological well-being seems inconsistent. Further, evidence suggests that individualism indeed is associated with suicidal phenomena, but the studies underlying this conclusion are of weak design, either being ecologic or single-nation studies. Hence, it is unwarranted to assume cross-cultural generality from these. Considering the inconclusive nature of research findings in this field, we designed the present study to explore the associations of individualistic-collectivistic value orientations with suicidal behavior, attitudes, and psychological distress in university students from 12 countries. From a methodological perspective, cross-national comparative studies require comparable samples. We assume that university students are similar in important aspects, such as their age, educational level, or cognitive abilities, and, to some extent, media exposure. Besides, collecting data with university students is a convenient way of getting sufficient data. Therefore, we have chosen to test our hypotheses in university students. Specifically, we tested the following five hypotheses:

1. Individualistic values are associated with more suicidal behavior and psychological distress, whereas collectivistic values with less suicidal behavior and psychological distress.
2. The prevailing culture (individualistic vs. collectivistic) in the investigated countries moderates the associations of individualistic and collectivistic values with suicidal behavior and psychological distress.
3. Individualistic values are associated with more permissive attitudes to suicide, whereas collectivistic values with less permissive attitudes to suicide.
4. Collectivistic values are associated with more socially accepting and helping attitudes to suicidal persons, whereas individualistic values with less socially accepting and helping attitudes to suicidal persons.
5. Across the investigated countries, the prevalence of completed suicide (i.e., national suicide rates) moderates attitudes towards suicide and towards suicidal persons.

## Materials and Methods

### Participants

A total of 5,572 (55.3% women) university students (age *M* = 22.1, *SD* = 3.5 years) from 12 countries volunteered to participate in the study. Samples originated from countries belonging to four culture zones (58): 1.) the Confucian (China *n* = 627; Japan *n* = 246), 2.) the Islamic (Iran *n* = 1000; Jordan *n* = 436; Palestine (West Bank) *n* = 358; Saudi Arabia *n* = 413; Turkey *n* = 497; Tunisia *n* = 484), 3.) the English-speaking (UK *n* = 150; USA *n* = 239), and 4.) the Catholic zone (Austria *n* = 627; Italy *n* = 471). Across countries, participants were recruited from one public university, with the exception of Jordan and Palestine (with recruitment at two public universities).

Across countries, the distributions of participants’ gender (χ^2^ = 294.56, *df* = 11, *p* < .001) and age [*F*(11, 5407) = 105.61, *p* < .001] differed significantly (see **Table 1**). Overall, samples were slightly skewed towards female participants (55.3% women). The Japanese and Saudi Arabian samples showed a surplus of men. The youngest sample was from the United States, and the oldest one from the United Kingdom. There were significant sample differences regarding participants’ stated sibship size, with the samples from Jordan and Palestine reporting the highest, and the sample from China reporting the lowest number of siblings. Further details of the sociodemographic characteristics of the participants and study procedural details, see Eskin et al. (59).

**Table 1:** Descriptive statistics of individualism and collectivism factor scores, suicidal ideation, suicide attempts, and GHQ-12 scores per country.

Country	*n*	%Women	Age *M* (*SD*)	Individualism *M* (*SD*)	Collectivism *M* (*SD*)	Suicidal ideation			Suicide attempt		GHQ-12 score		
						Life-time	Last 12 months	Current	Life-time	Last 12 months	≥ 3	≥ 4	≥ 5
Austria	627	55%	22.81 (3.37)	-0.30 (0.65)	1.17 (0.74)	48%	16%	6%	3%	<1%	38%	29%	22%
China	651	52%	21.47 (2.12)	0.11 (0.84)	-0.95 (0.73)	22%	7%	2%	4%	1%	35%	28%	21%
Iran	1000	60%	22.43 (3.93)	0.15 (1.07)	0.61 (0.77)	30%	15%	6%	5%	4%	45%	37%	30%
Italy	471	52%	23.29 (3.31)	-1.26 (0.98)	0.01 (0.75)	19%	4%	2%	3%	<1%	43%	29%	23%
Japan	246	33%	20.98 (2.21)	-0.24 (0.62)	-0.41 (0.68)	26%	10%	1%	3%	<1%	65%	55%	48%
Jordan	436	59%	21.10 (1.66)	-0.09 (1.03)	-0.79 (0.76)	22%	18%	14%	16%	15%	54%	47%	40%
Palestine	358	60%	20.83 (2.49)	0.37 (1.04)	-0.16 (0.80)	22%	16%	6%	12%	6%	69%	57%	47%
Saudi Arabia	413	30%	24.98 (3.50)	-0.06 (1.11)	-0.64 (0.92)	18%	10%	11%	10%	9%	79%	70%	61%
Tunisia	484	77%	21.47 (1.90)	0.67 (0.69)	0.20 (0.72)	21%	9%	3%	5%	1%	64%	52%	43%
Turkey	497	63%	20.57 (1.82)	0.28 (0.62)	0.02 (0.78)	24%	9%	3%	9%	2%	60%	49%	40%
United Kingdom	150	69%	26.93 (8.02)	0.06 (0.67)	-0.13 (0.74)	39%	15%	3%	7%	3%	NA	NA	NA
United States	239	51%	19.93 (3.89)	0.29 (0.71)	-0.23 (0.70)	31%	10%	< 1%	3%	0%	32%	25%	14%

GHQ-12, 12-item General Health Questionnaire.

### Materials

All data were collected through self-administered questionnaire forms, which included items about nonfatal suicidal behavior, religious affiliation and strength of religious belief, attitudes towards suicide and towards suicidal individuals, and individualistic-collectivistic value orientations, alongside a measure of psychological distress. The prevalence of nonfatal suicidal behavior and psychological distress, attitudes towards suicide and suicidal persons, and the associations of religion with suicidal behavior and attitudes and psychological distress have been reported elsewhere (59–61). The focus of the present account is the associations of individualistic-collectivistic value orientations with suicidal behavior, suicidal attitudes, and psychological distress.

#### Demographics

Participants reported their gender, age, and number of siblings.

#### Individualism-Collectivism

Based on their face and content validity, five items each were selected (22) from the Turkish version (62) of the INDCOL scale (Individualism and Collectivism scale) (63) for the assessment of individualism and collectivism. The five items tapping individualism were: 1.) I rely on myself most of the time; I rarely rely on others; 2.) I would rather depend on myself than others; 3.) Competition is the law of nature; 4.) Winning is everything; 5.) Being a unique individual is important to me. The five items tapping collectivism were: 1.) I like sharing little things with others; 2.) It is important to me that I respect the decisions made by my others; 3.) My happiness depends very much on the happiness of those around me; 4.) I would feel proud, if another person gets recognition; 5.) Group members should stick together, no matter what sacrifices are required. Whereas this shortened INDCOL scale was administered in the respective national language version in Austria, China, Iran, Italy, Japan, Turkey, the United Kingdom, and the United States, the English version was used in Jordan, Palestine, Saudi Arabia, and Tunisia. Participants responded to the INDCOL items on 5-point Likert-type scales, ranging from 1 = “completely disagree” to 5 = “completely agree”. The internal consistency (Cronbach α) for the individualism scale was.61 and.62 for the collectivism scale. For analysis, factor scores were used (see *Statistical Analysis* subsection).

#### Suicidal Behavior

Five questions queried past and current suicidal behavior, with response alternatives Yes = 1 vs. No = 0. These questions were: 1.) Have you ever thought of killing yourself? 2.) Have you, during the past 12-months, thought of killing yourself? 3.) Do you have thoughts of killing yourself right now? 4.) Have you ever made an attempt to kill yourself? 5.) Have you, during the past 12-months, made an attempt to kill yourself?

The scores of participants who responded affirmatively to at least one (or more) of the first three questions were dichotomized into the categories having suicidal ideation (vs. not), and the scores of participants who responded affirmatively to one (or both) of the questions 4 and 5 were dichotomized into the categories having attempted suicide (vs. not).

#### Psychological Distress

The 12-item General Health Questionnaire (GHQ-12) (64) was administered to assess psychological distress. Reliability and validity of the GHQ-12 have been established (65). The standard scoring method (of 0-0-1-1) was applied, i.e., a score of 0 is assigned to the first two low-stress response alternatives, and a score of 1 is given to the two high-stress response alternatives. This method yields individual scores ranging from 0 to 12. The Cronbach’s α for the GHQ-12 was.87, with item-total correlations ranging from.45 to.62. Previous research (65) has evidenced a variety of cut-off points for the GHQ-12, ranging from a low of 2 to a high of 4, across 15 centers. We thus applied three cut-off points (GHQ-12 ≥ 3, 4, or 5). The GHQ-12 was not administered in the United Kingdom.

#### Attitudes Towards Suicide

Eskin’s 24-item Attitudes Towards Suicide Scale (E-ATSS) (22, 60, 66), with 5-point Likert-type response options, ranging from 1 = “completely disagree” to 5 = “completely agree” was used to measure participants’ attitudes towards suicide. Principal component analysis with varimax rotation extracted six factors: 1.) Acceptability of suicide (eight items, α =.91); 2.) Punishment after death (five items, α =.93); 3.) Suicide as a sign of mental illness (three items, α =.94); 4.) Communicating psychological problems (four items, α =.79); 5.) Hiding suicidal behavior (two items, α =.83); and 6.) Open reporting and discussion of suicide (two items, α =.62) that accounted for 73.1% of the total variance. Scale scores were computed by summing up the items of a factor, divided by the number of items. Thus, scale scores ranged from 1 to 5, with higher scores indicating higher levels of factor content.

#### Attitudes Towards Suicidal Persons

Eskin’s Social Reactions to Suicidal Persons Scale (E-SRSPS) was used to measure social reactions to a suicidal peer. The introductory part of this instrument comprises a short description of “an imagined suicidal close friend” who decides to kill him/herself and shares this information with the respondent. By means of 20 possible reactions to this friend, participants were asked how they would react or feel on 5-point Likert-type scales ranging from 1 = “completely disagree” to 5 = “completely agree” (22, 60, 66). A principal component analysis with varimax rotation extracted four factors: 1.) Social acceptance (six items, α =.90); 2.) Helping a suicidal friend (six items, α =.83); 3.) Disapproval of suicidal disclosure (five items, α =.77); and 4.) Emotional involvement (three items, α =.63) that accounted for 60.7% of the total variance. Scale scores were computed by summing up the items under a factor, divided by the number of items. Thus, scale scores ranged from 1 to 5, with higher scores indicating higher levels of factor content.

### Procedure

The principal investigator (author ME) selected the questionnaire ensemble and invited researchers *via* e-mail to join the study. All participating researchers were university-based, collecting their dataset at their academic institution. For the Jordan and Palestine study sites, data were additionally collected at a second university. On the first page of the questionnaire packet, participants were told that the study was anonymous from the outset and participation entirely voluntary. Contact information of the respective study-site investigator was provided on the first survey page, so that participants could get in touch, for asking any study-related questions.

All researchers were requested to undertake data collection only after receipt of approval from relevant institutional review boards. Except for Austria, where such an approval formally was not necessary, according to the relevant national legal requirements and regulations, approval was obtained at all study sites. In the United Kingdom, data collection was stopped by the ethics committee due to one member’s concerns over possible distress effects of the suicide-related questions. Only the Jordanian researchers reported legal sanctions against suicidal behavior. According to the Jordanian Penal Code, “the person who attempts suicide will be punished by imprisonment from 3 months to 2 years”.

### Data-Analytic Strategy

#### Cross-National Measurement Equivalence

In order to ensure that measured scores were comparable between countries and to obtain scores on a common scale for all countries, the INDCOL, E-ATSS, and E-SRSPS items were subjected to tests of cross-national measurement equivalence, utilizing methods of multigroup confirmatory factor analysis. To make these analyses computationally feasible, data from the United Kingdom (for which only a relatively small sample was available) and the United States were merged. Also, for some of the E-ATSS and E-SRSPS subscale analyses, data from China and Japan had to be merged. Mplus 8.2 was used for tests of measurement equivalence, treating the items as ordered categorical variables by utilizing the WLSMV estimator. This is, in this factor analytic context, comparable to fitting Samejima’s graded response model (67) to the data, wherein each item is described by a single discrimination parameter (item loading) and *m*—1 difficulty parameters for its *m* response options (item thresholds) [see also (68)].

Separately for all scales and subscales, we tested the data for cross-national configural invariance (i.e., whether all respective scale or subscale items loaded onto a single latent factor across all countries) and full measurement invariance (i.e., whether all loadings and thresholds of items within a scale or subscale were the same across all countries). Equivalence of item parameters across countries was then relaxed in an iterative procedure, where necessary, to arrive at a final model of partial measurement invariance that showed an acceptable data fit. Partial measurement invariance indicates that the parameters of some, but not all, items were equal across groups. Partial measurement invariance may still allow for meaningful comparisons between groups (69); however, comparisons need to be made with caution. Item parameters were freed for single countries or set to equivalent values for groups of countries. In this procedure, item loadings and thresholds were freed in tandem, because both types of item parameters conjointly define the regression curve of the item on the latent trait. For the final models, factor scores were then extracted and used in subsequent analysis. All E-ATSS and E-SRSPS subscales were found to be fully invariant, except the E-SRSPS subscales Helping a suicidal friend, for which partial invariance was observed, and Disapproval of suicidal disclosure, for which one item was removed to first achieve configural invariance (see *Results*). As the results of the multilevel analyses did not critically depend on the use of factor scores for the fully invariant scales, results based on scale scores are presented for simplicity. For the partially invariant E-SRSPS subscale, factor and scale scores correlated with *r* =.93 (*p* < .001) and the results of the multilevel analyses did not critically depend on the use of factor scores. Hence, also in this case results based on scale scores are presented for simplicity.

Model fit was assessed with the comparative fit index (CFI), the Tucker-Lewis index (TLI), and the standardized root mean square residual (SRMR), utilizing the benchmarks of Hu and Bentler (70) (CFI/TLI: good fit: ≥.95, acceptable fit: ≥.90) and Schermelleh-Engel et al. (71) (SRMR: good fit: < .05, acceptable fit: < .10). Values of the root mean square error of approximation (RMSEA) were not used, as the models were fitted on a large number of groups (> 10) with only a few indicators (e.g., five items each for individualism and collectivism). In the multigroup context, RMSEA values are estimated from the square root of the weighted average of the sample-based discrepancies, divided by the average degrees of freedom (*df*) per sample (72), not the overall *df*. This resulted in the present study in small average *df*s (~ 5), especially in the configural invariance analyses. Yet, in models with small *df*, RMSEA values are inflated, rendering them uninformative for the evaluation of model fit (73). Similarly, we report chi-square values of model fit, but do not interpret them with regards to significance as chi-square values are inflated in large samples (71).

CFI and TLI compare the fit of the investigated model against a null model, which assumes no latent variables and uses the identity matrix as variance-covariance matrix. The CFI compares the chi-square to *df* differences between the null and the investigated model, whereas the TLI the respective chi-square to *df* ratios. Under maximum likelihood (ML) estimation, this entails smaller TLI than CFI values (which is consistent with the interpretation that the TLI more strongly penalizes model complexity than the CFI). However, under WLSMV, the *df* are estimated from the data and are not determined by the difference in the number of observed to estimated parameters, see (74). Compared to the null model, this led in many of the multigroup analyses of the present study to especially small *df* in less restrictive models (e.g., configural invariance models, which estimate large numbers of parameters), and especially large *df* in more restrictive models (e.g., full invariance models, which estimate only relatively few parameters). This either (less restrictive models) excessively disadvantaged TLI to CFI values, or (more restrictive models) also CFI to TLI values (a case that cannot similarly arise under ML).

Against this background, model fit was considered acceptable, if at least one of the two goodness-of-fit indices (CFI, TLI) and the SRMR (an absolute badness-of-fit index, which assesses the standardized difference between the observed and predicted correlations) indicated acceptable model fit.

#### Associations With Suicidal Ideation, Suicide Attempts, and Psychological Distress

In order to account for the clustered nature of the data, multilevel (more precisely, two-level) regression models were then applied to investigate the associations of individualism and collectivism factor scores with suicidal ideation, suicide attempts, and psychological distress (as the level-1 predictors), using country as a cluster variable (i.e., level-2 predictors). This utilization of multilevel models allowed the modeling and testing of regression slopes on the mean level (i.e., averaged across all countries) for statistical significance, and further to investigate the variability of individual regression slopes (and intercepts) across countries. These models were further utilized to examine possible effects (i.e., cross-level interactions) of level-2 predictors (the country level) on the regression slopes and intercepts of the level-1 predictors (the individual level).

Mplus 8.2 was again used for analysis, using numerical integration and robust methods (MLR) for the estimation of standard errors. All models included individualism and collectivism factor scores as level-1 predictors (controlling also for participant sex and age, see below), testing their linear, but in a second step also their quadratic, associations with the various outcomes separately for each outcome. By including quadratic terms, we controlled and tested for the possible non-linearity of the associations of individualism and collectivism with the outcome variables. Outcomes were modeled as binary variables. Hence, the fitted models were multilevel logistic regression models. For these models, we report unstandardized slope coefficients (on the logit scale). These appear to fit better the present context of multilevel modeling, which directly deals with the variation of slopes and intercepts on this scale. Odds ratios may be obtained from the reported slope parameters by exponentiation.

Model building proceeded in three steps: in the first step, intercepts and slopes on level 1 were modeled as random effects (i.e., they were allowed to vary between countries), estimating the covariance between intercepts and slopes freely from the data. If the variability of an individual slope parameter was not significant [*p* > .05; instead of the Wald test the more powerful likelihood ratio test, comparing models with and without this variance parameter and the covariance, was used here; see ref. (75), pp. 98–99], the respective slope parameters were in a second step modeled as fixed effects, in order to arrive at more parsimonious final models. Analyses controlled for participant sex and age by including them as further level-1 predictors in the models. We report on the effects of sex and age in detail, where their effects appeared to be significant (*p* < .05; based on the Wald test). For the effect tests of individualism and collectivism, sequential Bonferroni corrections were applied (using an overall α of 5%) to control for the accumulation of type I errors. The results of these analyses were used to test Hypothesis 1.

In a third step, potential cross-level interactions of culture (level 2) with random slopes (level 1), were investigated for the above models. Countries included in the study were classified into individualistic and collectivistic categories on the basis of their aggregated country individualism scores (6). Accordingly, Austria, Italy, the United Kingdom and the United States were grouped as individualistic countries. Asian and Middle Eastern collectivisms may involve different cultural patterns. Therefore, two groups of collectivistic cultures were created. China and Japan were grouped together and termed as Asian collectivistic cultures. Likewise, Jordan, Iran, Palestine, Saudi Arabia, Tunisia, and Turkey were grouped together and termed as Muslim collectivistic cultures. We created two level-2 dummy variables to indicate Asian and Muslim collectivistic cultures and used these as predictors of random slopes and random intercepts (which are of less interest here) on level 1. The results of these analyses were used to test Hypothesis 2.

#### Associations With Attitudes and Reactions Towards Suicide and Suicidality Factors

Multilevel models were used in a similar fashion as in Steps 1 and 2 of the foregoing analyses to investigate the associations of individualism and collectivism factor scores with the E-ATSS and E-SRSPS subscale scores. Outcomes were modeled as continuous variables in these analyses. Hence, fitted models resembled ordinary multilevel linear regression analyses. The results of these analyses were used to test the Hypotheses 3 and 4.

To test Hypothesis 5, we examined the associations of the country-level mean E-ATSS and E-SRSPS subscale scores with national suicide rates. For this goal, national suicide rate was used as a level-2 variable to predict random intercepts in these subscales in models without any focal level-1 predictors, but controlling for participant sex and age. The respective national suicide rates were taken from the World Health Organization (76). Palestine was excluded from this analysis, as no suicide rate was available for this unit of analysis.

Finally, similarly to the third step of analysis of the foregoing analyses, possible cross-level interactions of national suicide rate with random slopes (and intercepts) for the associations of individualism and collectivism with the E-ATSS and E-SRSPS subscale scores were investigated in an exploratory fashion.

## Results

### Cross-National Measurement Equivalence

All scales and subscales exhibited at least acceptable levels (with reference to either CFI and/or TLI and SRMR values) of configural invariance (see **Supplementary Materials**); i.e., every scale and subscale was essentially unidimensional in all investigated countries. Full measurement invariance could be assumed for the E-ATSS and E-SRSPS subscales (except Helping a suicidal friend) as well. The individualism and collectivism scales exhibited only partial measurement invariance. The final partial measurement invariance models for these two scales (and of Helping a suicidal friend) were obtained by relaxing the equivalence of item parameters between countries or groups of countries in a stepwise fashion until an acceptable fit was achieved.

Means and standard deviations of the individualism and collectivism factor scores of the partial measurement models in the investigated countries are displayed in **Table 1** (standardized across all countries to yield a grand mean of 0 and a variance of 1; thereby, predictors were also grand-mean-centered for the subsequent multilevel regression analyses). As can be seen, Muslim countries like Palestine, Tunisia, and Turkey, but also the USA, had the highest scores in individualism; Italy, Austria, and Japan had the lowest scores. For collectivism, Austria and Iran had the highest scores; China, Jordan, and Saudi Arabia had the lowest scores. As only partial measurement invariance was achieved, direct comparisons between countries need to be made with caution, however. Across countries, individualism and collectivism were weakly interrelated (*r* =.13, *p* < .001). Within-country correlations were particularly high for Saudi Arabia (*r* =.78, *p* < .001) and Palestine (*r* =.37, *p* < .001), but otherwise ranged from *r* = -.13 (Italy) to *r* =.29 (Jordan). Excluding Saudi Arabia from the further analyses did not substantially alter their results. Hence, the data from Saudi Arabia were included in all subsequent analyses.

### Suicidal Ideation, Suicide Attempts, and Psychological Distress (Hypothesis 1)

Descriptive statistics on suicidal ideation, suicide attempts, and psychological distress in the investigated countries are provided in **Table 1**. The results of the multilevel analyses are presented in **Table 2**. Mostly, linear associations of individualism with suicidal ideation and suicide attempts were not significant at the mean level (i.e., averaged across all countries); control variables participant sex and age did not affect these outcomes, except that suicidal ideation in the last 12 months was more likely reported by younger than older individuals (*p* =.048). Higher individualism appeared to be linearly associated only with less current suicidal ideation and a lower likelihood of a suicide attempt in the last 12 months across all samples. However, there were also quadratic associations of individualism with suicidal ideation and suicide attempts. Overall, both lower and higher than average individualism scores were associated with a higher likelihood for life-time suicidal ideation, suicidal ideation in the last 12 months, and any suicidal ideation. For suicide attempts in all investigated periods, individuals low in individualism, compared to individuals with intermediate or high scores, also had an overall higher likelihood for reporting an attempt.

**Table 2:** Associations of individualism and collectivism with suicidal ideation, suicide attempts, and psychological distress.

Outcome variables	Slope coefficients		Random-effects variance estimates		
	Individualism	Collectivism	Intercept	Slope Individualism	Slope Collectivism
Suicidal ideation					
Life-time	-0.016 (0.049)	-0.136 (0.071)	0.212 (0.118)	—	0.041 (0.017)*
	0.072 (0.032)*	0.022 (0.046)		—	—
Last 12 months	-0.009 (0.098)	-0.241 (0.082)**	0.263 (0.101)**	0.083 (0.035)*	—
	0.084 (0.020)***	0.045 (0.036)		—	—
Current	-0.273 (0.126)*	-0.251 (0.122)*	0.804 (0.289)**	—	—
	0.031 (0.043)	0.033 (0.044)		—	—
Any	-0.008 (0.047)	-0.149 (0.060)*	0.450 (0.279)	—	—
	0.070 (0.030)*	-0.002 (0.034)		—	—
Suicide attempt					
Life-time	-0.207 (0.146)	-0.216 (0.105)*	0.381 (0.164)*	—	—
	0.092 (0.031)**	0.058 (0.029)*		—	—
Last 12 months	-0.295 (0.129)*	-0.340 (0.195)	2.073 (0.858)*	—	—
	0.156 (0.040)***	-0.032 (0.051)		—	—
Any	-0.238 (0.126)	-0.226 (0.097)*	0.543 (0.221)*	—	—
	0.125 (0.021)***	0.007 (0.042)		—	—
Psychological distress					
GHQ-12 ≥ 3	-0.053 (0.049)	0.014 (0.069)	0.445 (0.149)**	0.038 (0.016)*	—
	0.087 (0.031)**	0.023 (0.048)		—	—
GHQ-12 ≥ 4	-0.116 (0.062)	-0.046 (0.068)	0.404 (0.128)	—	—
	0.062 (0.034)	0.046 (0.048)		—	—
GHQ-12 ≥ 5	-0.120 (0.028)***	0.020 (0.059)	0.471 (0.165)**	0.063 (0.022)**	—
	0.091 (0.038)*	0.026 (0.037)		—	—

Numbers are parameter estimates (standard errors in parentheses). Slope coefficients for linear terms are presented in the first line, and for quadratic terms in the second line for each outcome. Slopes were modeled as random effects, where respective variance estimates are provided, and as fixed effects otherwise. Displayed significance levels are based on Wald tests for slope coefficients, and likelihood ratio tests for random-effects variance estimates (confidence intervals for the variance estimates may not reliably reproduce the significance of these more powerful tests). Participant sex and age (not shown) were used as level-1 control variables for all outcomes, see main text for further details. *p < .05, **p < .01, ***p < .001.

In contrast, higher collectivism appeared to be linearly associated with less suicidal ideation in the last 12 months, current suicidal ideation, and any suicidal ideation; and with a lower likelihood of a life-time suicide attempt and any suicide attempt (either life-time or in the last 12 months). For life-time suicide attempts there was also a quadratic association with collectivism, such that (in combination with the linear effect) individuals low in collectivism, compared to individuals with intermediate or high scores, had a higher likelihood to report an attempt. Slopes of the linear, but not the quadratic terms, varied somewhat between countries for life-time suicidal ideation and suicidal ideation in the last 12 months [see *Cross-Level Interactions With Culture (Hypothesis 2*)].

Controlling for multiple testing (using sequential Bonferroni correction) with regards to the 16 effect tests for linear and quadratic associations of individualism and collectivism with suicidal ideation, the quadratic association of individualism (*p* < .001) and the linear association of collectivism (*p* =.003) with suicidal ideation in the past 12 months remained significant (overall α = 5%).

Similarly controlling for multiple testing with regards to the respective 12 effect test for suicide attempts, all quadratic associations of individualism with life-time suicide attempts (*p* =.003), suicide attempts in the last 12 months (*p* < .001), and any suicide attempts (*p* < .001) retained their significance (overall α = 5%).

Individualism also exhibited a quadratic association with psychological distress (cut-offs 3 and 5), such that individuals with either low or high scores had a higher likelihood of crossing the cut-off than individuals with intermediate scores. Additionally, there was a negative linear association of individualism with psychological distress (cut-off 5), indicating that individuals with high scores were somewhat less likely to cross this cut-off than individuals with low scores. Overall, women were more likely to report psychological distress for all cut-offs (*p*s ≤.041) than men; also, for the cut-off of 3, younger individuals more likely reported psychological distress than older ones (*p* =.016). Cross-country variability was apparent with regards to the linear associations of individualism with psychological distress.

Controlling for multiple testing with regards to the 12 effect tests of individualism and collectivism with psychological distress, all associations but the quadratic association of individualism with psychological distress (cut-off 5) retained their significance (overall α = 5%).

### Cross-Level Interactions With Culture (Hypothesis 2)

#### Suicidal Ideation and Suicide Attempts

The associations of predictors with outcomes on the level 1 varied between cultures. Among individualistic cultures, collectivism on average was negatively linearly associated with life-time suicidal ideation (**Table 3**). Thus, controlling for individualism, higher collectivism appeared to be protective against suicidal ideation among individualistic cultures, whilst not among Asian and Muslim collectivistic cultures.

**Table 3:** Mean slopes of individualism and collectivism in the investigated cultures.

Outcome variable: Predictor	Culture			Simple contrasts
	(1) Individualistic	(2) Asian collectivistic	(3) Muslim collectivistic	
Suicidal ideation				
Life-time: Collectivism	-0.305 (0.031)***	0.113 (0.106)	-0.106 (0.118)	2 > 1
Last 12 months: Individualism	0.241 (0.123)	0.457 (0.088)***	-0.225 (0.094)*	1,2 > 3
Psychological distress				
GHQ-12 ≥ 3: Individualism	0.157 (0.039)***	-0.014 (0.057)	-0.148 (0.047)**	1 > 2 > 3
GHQ-12 ≥ 5: Individualism	0.085 (0.088)	-0.021 (0.052)	-0.140 (0.059)*	1,2 > 3

Individualistic cultures = Austria, Italy, UK, USA; Asian collectivistic cultures = China, Japan; Muslim collectivistic cultures = Iran, Jordan, Palestine, Saudi Arabia, Tunisia, Turkey. Numbers are parameter estimates (standard errors in parentheses). “Simple contrasts” lists significant differences (p < .05) in the mean slopes between cultures. *p < .05, **p < .01, ***p < .001. GHQ-12, 12-item General Health Questionnaire.

Concerning the linear associations of individualism with suicidal ideation in the last 12 months, the mean slopes among Muslim collectivistic cultures were significantly negative, whereas significantly positive among Asian collectivistic cultures (**Table 3**). Combined with the overall quadratic effect of individualism, this indicated that in Asian collectivistic cultures specifically individuals high in individualism, compared to individuals with low or intermediate scores, had a higher likelihood to report suicidal ideation in the last 12 months. In contrast, in Muslim collectivistic cultures, specifically individuals low in individualism had a higher likelihood to report suicidal ideation in the last 12 months than individuals with intermediate or high scores. Thus, specifically high individualism appeared to be a risk factor for suicidal ideation in Asian collectivistic cultures, whereas low individualism in Muslim collectivistic cultures.

#### Psychological Distress

For a cut-off of 3, the slope of the linear association of individualism with psychological distress was significantly positive for individualistic cultures and significantly negative for Muslim collectivistic cultures. Combined with the overall quadratic effect of individualism, this indicated that the likelihood of reporting psychological distress was elevated for individuals low in individualism (compared to individuals with high or intermediate scores) in Muslim collectivistic cultures, but elevated for individuals high in individualism in individualistic cultures. A similar trend was apparent in Muslim collectivistic cultures for a cut-off of 5.

### Attitudes and Reactions Towards Suicide and Suicidality Factors (Hypotheses 3 and 4)

Descriptive statistics on attitudes and social reactions towards suicide and suicidality factors in the investigated countries are provided in the **Supplementary Materials**.

In the following, we report only on associations of individualism and collectivism with attitudes (**Table 4**) which were significant after controlling for multiple testing (as above; 24 tests, overall α = 5%). At the mean level (averaged across all countries), higher collectivism was linearly negatively associated with acceptability of suicide and positive with punishment after death and communicating psychological problems. Individualism was linearly positively associated with hiding suicidal behavior and quadratically with suicide as a sign of mental illness, such that a positive association at the low range of individualism scores levelled off for intermediate and high scores (i.e., there was no further increase in the range of intermediate and high scores). Associations were mostly stronger with collectivism than with individualism. Regarding the control variables, men overall had higher scores than women with regards to acceptability of suicide (*p* < .001) and hiding suicidal behavior (*p* =.001). Younger participants had higher scores in punishment after death than older participants (*p* < .001).

**Table 4:** Associations of individualism and collectivism with attitudes and reactions towards suicide and suicidality factors.

Outcome variables	Slope coefficients		Random-effects variance estimates		
	Individualism	Collectivism	Intercept	Slope Individualism	Slope Collectivism
Attitudes towards suicide factors					
Acceptability of suicide	0.031 (0.035)	-0.166 (0.016)***	0.138 (0.051)**	0.011 (0.004)***	—
	0.018 (0.012)	0.013 (0.010)		—	—
Punishment after death	0.058 (0.045)	0.093 (0.036)**	0.737 (0.089)***	—	0.020 (0.007)***
	-0.028 (0.020)	-0.049 (0.022)*		—	—
Suicide as a sign of mental illness	0.057 (0.039)	0.087 (0.051)	0.194 (0.046)***	—	0.027 (0.009)***
	-0.043 (0.012)***	-0.019 (0.015)		—	—
Communicating psychological problems	0.048 (0.023)*	0.230 (0.026)***	0.043 (0.010)***	—	0.007 (0.004)***
	-0.029 (0.017)	-0.056 (0.024)*		—	—
Hiding suicidal behavior	0.088 (0.033)**	-0.043 (0.047)	0.159 (0.037)***	0.007 (0.002)***	—
	-0.001 (0.024)	-0.039 (0.027)			
Open reporting and discussion of suicide	0.045 (0.019)*	0.070 (0.027)*	0.161 (0.068)*	—	—
	-0.029 (0.022)	0.012 (0.014)		—	—
Reactions to suicidality factors					
Social acceptance	0.103 (0.024)***	0.218 (0.024)***	0.045 (0.015)**	0.006 (0.002)***	—
	-0.030 (0.016)	-0.019 (0.012)		—	—
Helping a suicidal friend	0.081 (0.021)***	0.191 (0.019)***	0.022 (0.009)*	—	—
	-0.033 (0.021)	-0.023 (0.015)		—	—
Disapproval of suicidal disclosure	0.049 (0.016)**	-0.014 (0.024)	0.100 (0.028)***	0.002 (0.001)**	—
	-0.030 (0.008)***	-0.036 (0.012)**		—	—
Emotional involvement	0.094 (0.020)***	0.115 (0.025)***	0.176 (0.048)***	—	—
	-0.022 (0.015)	-0.018 (0.016)		—	—

Numbers are parameter estimates (standard errors in parentheses). Slopes were modeled as random effects, where respective variance estimates are provided, and as fixed effects otherwise. Displayed significance levels are based on Wald tests for slope coefficients, and likelihood ratio tests for random-effects variance estimates (confidence intervals for the variance estimates may not reliably reproduce the significance of these more powerful tests). Participant sex and age (not shown) were used as level-1 control variables for all outcomes, see main text for further details. *p < .05, **p < .01, ***p < .001.

Concerning reactions to suicidality factors (**Table 4**; all associations remained significant after controlling for multiple testing; 16 tests, overall α = 5%), individualism was at the mean level linearly positively associated with social acceptance, helping a suicidal friend, and emotional involvement. Its association with disapproval of suicidal disclosure was overall nonlinear (combining the linear and quadratic associations), such that a positive association at the low range of individualism scores levelled off (i.e., there was no increase) for intermediate and high scores. Collectivism was at the mean level linearly positively associated with social acceptance, helping a suicidal friend, and emotional involvement. Its nonlinear association with disapproval of suicidal disclosure was such that, controlling for individualism, disapproval decreased for both high and low scores of collectivism. Regarding the control variables, women had overall higher scores than men in social acceptance (*p* < .001), helping a suicidal friend (*p* =.002), and emotional involvement (*p* =.003). Younger participants had higher scores in disapproval of suicidal disclosure than older participants (*p* =.002).

### Moderating Effects of National Suicide Rate (Hypothesis 5)

Suicide rates mostly did not moderate the mean E-ATSS and E-SRSPS subscale scores at the country level (*p*s ≥.075). However, moderating effects were observed for punishment after death (slope = -0.100, *SE* = 0.029, *p* < .001), suicide as a sign of mental illness (slope = -0.051, *SE* = 0.021, *p* =.013), and emotional involvement (slope = -0.042, *SE* = 0.008, *p* < .001). Mean scores in these scales (controlling for participant sex and age) were lower in countries with higher national suicide rates.

### Cross-Level Interactions With National Suicide Rate (Exploratory Analysis)

**Figure 1:**
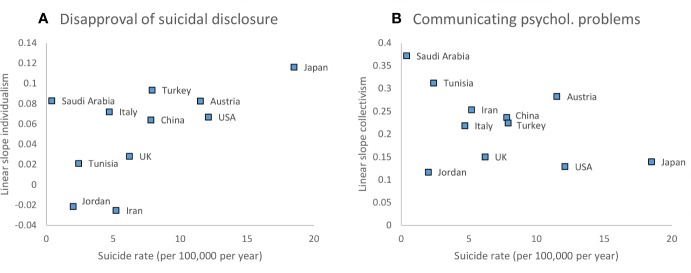
Slope coefficients (on the ordinate) by national suicide rate (on the abscissa) of **(A)** individualism for disapproval of suicidal disclosure and **(B)** collectivism for communicating psychological problems.

National suicide rates did not account for any variability in the linear slopes of individualism and collectivism for most dependent variables (*p*s ≥.077). However, suicide rates moderated the linear association of individualism with disapproval of suicidal disclosure (slope = 0.005, *SE* = 0.002, *p* =.013): linear slopes were stronger positive in countries with higher suicide rates (**Figure 1A**). Combined with the overall quadratic association [see *Attitudes and Reactions Towards Suicide and Suicidality Factors (Hypotheses 3 and 4)*], this indicated that increases of disapproval with individualism in the lower score range actually reverted for higher score ranges in countries with lower suicide rates.

Also, suicide rates moderated the linear association of collectivism with communicating psychological problems (slope = -0.010, *SE* = 0.005, *p* =.047): linear slopes were stronger positive in countries with lower suicide rates (**Figure 1B**).

## Discussion

Research indicates that the individualism-collectivism dimension provides important sources of information for both intersocietal and interindividual similarities and differences. Therefore, the present study investigated the associations of individualistic and collectivistic value orientations with nonfatal suicidal behavior, attitudes towards suicide and suicidality, and psychological distress in student samples from 12 countries by testing five research hypotheses. The findings from these yielded interesting features, which may shed light on the relations of individualism-collectivism to suicidal behavior, attitudes towards suicide and suicidal persons, and psychological distress in young adults enrolled in higher-education institutions.

It is noteworthy that while the individualism scores were highest in traditionally collectivistic countries like Palestine, Tunisia, and Turkey, together with the USA, the lowest individualism scores were noted in traditionally individualistic countries, such as Austria and Italy, together with Japan. Whereas Austria and Iran had the highest collectivism scores, China, Jordan and Saudi Arabia had the lowest collectivism scores. It seems that young adults in individualistic countries seek communion with others, while their age mates in collectivistic countries seek to assert their individuality, which is in line with the Arab Spring uprisings in Arab countries (77) and the Gezi protests in Istanbul, Turkey (78). Another reason for high individualism scores in traditionally collectivistic countries might be related to the item content of the utilized scale. Items in the individualism scale mostly dealt with competition. Due to the limited resources in their countries, participants might have specifically endorsed competition, which, however, is only one aspect of individualism, not its whole content. Also, the individualism and collectivism scales exhibited only partial measurement invariance in the current study. Conclusions thus have to be made with caution. Fully invariant scales are still needed.

The scientific evidence indicates that, compared to people with predominantly collectivistic values, people with individualistic values report more independent self-concepts and context-independent cognitive processing styles, and lower relationality and dispositional or internal attributions (10–15). It has previously been argued (22) that individuals with individualistic value orientations, when experiencing negative life circumstances, may feel personal responsibility for the situation and may blame themselves for what happened which, in turn, may result in feelings of anger, unhappiness, and hopelessness during times of personal crisis. Such an attribution process may have dire consequences for the individual, when coupled with insufficient social support. Conversely, when experiencing negative life circumstances, individuals with high collectivistic value orientations may attribute responsibility to others or to situations. This, in turn, may well mitigate the impact of anger, unhappiness, and hopelessness, which are the predominating cognitive-affective states in suicidal developments (79).

Based on research findings related to the differences between individualism and collectivism, our first hypothesis predicted that individualistic values would be associated with more suicidal behavior and psychological distress, whereas collectivistic values would be associated with less suicidal behavior and psychological distress. The results provided mixed support for this prediction. In line with findings from Eskin (22) and Scott et al. (42), collectivism was associated with less life-time suicidal ideation, but unlike these previous findings, the shape of the associations of individualism with suicidal ideation within a 12-month period, and of suicide attempts appeared to be “u”-shaped: Both individuals with high and low, but not intermediate, levels of individualism had a higher likelihood of suicidal ideation and behavior. Individualism was also associated with a higher likelihood of psychological distress (linearly with a higher scale cut-off; again u-shaped with a lower cut-off), controlling for participant sex and age. However, our results demonstrated some variability in the slopes of individualism and collectivism between countries. Thus, the associations of the two value orientations to suicidal ideation and psychological distress appear in part to be context-dependent, rather than being universal.

Individuality (agency, differentiation) and relatedness (communion, assimilation) are the two universal human needs (80–82). This duality of social needs corresponds well to the dichotomy of the individualism-collectivism dimension. Research shows that national cultures, with their specific value structures, differ from another or resemble each other with regards to their location on this cultural dimension (6). It is possible that some cultures, in line with their location on the individualism-collectivism dimension, might be better prepared for satisfying one social need over others. For instance, cultures on the individualistic pole may well be more prepared for gratifying the need for individuality, whereas those on the collectivistic pole are more prepared for gratifying the need for communion or relatedness. Thus, there is a discrepancy between country (or group) culture and individual need satisfaction. Previously, Caldwell-Harris and Ayçiçeği (47) have highlighted this as the personality-culture clash hypothesis.

On this background, our second hypothesis predicted that the prevailing culture (individualistic vs. collectivistic) in the investigated countries would moderate the associations of individualistic and collectivistic values with suicidal behavior and psychological distress. Our results yielded support for this idea. Collectivism on average was significantly negatively associated with life-time suicidal ideation in individualistic countries, but not in Asian and Muslim countries. In turn, whereas both individuals high and low in individualism were at risk for suicidal ideation in the last 12 months in individualistic countries, only individuals low in individualism where at risk in Muslim collectivistic countries, and only individuals high in individualism in Asian collectivistic countries. Patterns were somewhat different for psychological distress, where individuals at risk appeared to be again either situated at the low range of individualism (Muslim collectivistic countries), the high range (individualistic countries), or at both ends of the distribution (Asian collectivistic countries).

Individualism signifies independence, freedom, choice, personal responsibility, and competition (23, 24). The suicidal act frequently is seen as personal freedom and choice (25, 26). Consistent with this view, a majority of suicidal individuals seem not to seek professional or nonprofessional help (27, 28). Concurrently, perceived stigma, embarrassment, and self-reliance preferences are widely seen as important general barriers to help-seeking behaviors in younger individuals affected with mental health problems, including suicidality (29–31). Within such a value matrix, it is reasonable to assume that people with an individualistic world view will see suicide as personal freedom and choice, and subsequently they will hold higher levels of acceptance for suicide than those with a collectivistic world view. On these grounds, our third hypothesis anticipated that individualistic values would be associated with more permissive attitudes to suicide, whereas collectivistic values with less permissive attitudes. Consistent with prior related evidence (4, 5, 22, 38), our results indicate that collectivistic values are uniformly associated with less permissive attitudes to suicide, whereas individualistic values with a more stigmatized view of suicidal behavior. Yet, we found that the tendency to view suicide as a sign of mental illness increased only in the low score range of individualism, but not the intermediate and high ranges. Thus, our third hypothesis was confirmed.

Independence, freedom, and personal responsibility are core values in the concept of individualism, and persons with individualistic value orientations display context-independent information processing and dispositional attribution styles, compared to context-dependent and situational attribution style shown by those with collectivistic value orientations. Further, persons engaging in suicidal behavior are reluctant to seek help for reasons related to a belief in self-reliance, stigma, and a belief that nobody could help (83, 84). Thus, our fourth hypothesis predicted collectivistic values would be associated with more socially accepting/helping attitudes to suicidal persons, whereas individualistic values with less socially accepting/helping attitudes to suicidal persons. Like the findings from Eskin (22), the results obtained from this study produced some support for our fourth prediction. Both individualistic and collectivistic values were significantly associated with socially accepting attitudes to a suicidal peer, helping a suicidal friend, and emotional involvement. These associations were overall stronger for collectivism than for individualism. Yet, both individualistic and collectivistic values were also nonlinearly associated with disapproving attitudes to suicidal disclosure, but in slightly dissimilar fashions: Disapproving attitudes increased with increasing scores in the low score range of individualism and collectivism, but decreased only in the high score range of collectivism.

Research indicates that individuals in high-suicide-rate countries more strongly approve suicide than their counterparts in low-suicide-rate countries (5). Also, research indicates that people with a suicidal past (85) and people from regions with a high suicide rate (86) have less positive attitudes and lower intentions to help-seeking behaviors. In similar vein, Crowder and Kemmelmeier (87) showed that in regions with high suicide rates people are less likely to seek out psychiatric services for depression. Against this background, in our fifth hypothesis we tested whether national suicide rates are associated with attitudes towards suicide and suicidal persons in the countries investigated. The results revealed that participants in countries with higher suicide rates believed less that persons engaging in suicidal behavior would be punished after death and that suicide is a sign of mental illness; however, they also displayed less emotionally engaging reactions towards a suicidal peer than their counterparts. These results dovetail with insights from prior related comparative research (33, 34).

It is interesting to note that national suicide rates also moderated the linear associations of individualism with disapproval of suicidal disclosure and of collectivism with communicating psychological problems. Thus, the association of individualistic values with disapproval was more linear in countries with higher suicide rates, whereas more quadratic in countries with lower suicide rates. This implies that in countries with higher suicide rates only lower individualism was actually associated with less disapproval, whereas in countries with lower suicide rates both lower and higher individualism was associated with less disapproval of disclosure. Positive linear associations of collectivism with communicating psychological problems were stronger in countries with lower suicide rates. This might be indicative of the relative ease of activating the informal social support systems in these countries, which is in line with research demonstrating the protective functions of social support against suicidal behavior (88).

As an aside, the current results suggest that the E-ATSS and E-SRSPS subscales are readily applicable in cross-cultural research, as their items appeared to exhibit full measurement invariance in the herein investigated countries. This is further evidence of the good psychometric properties of these two scales (89). Reported means and standard deviations of the E-ATSS and E-SRSPS subscales in the herein investigated countries may be used as reference in future cross-cultural suicide research.

### Limitations

Although our results provide a variety of clues for a possibly causal involvement of individualism in self-reported suicidal behavior and psychological distress, for several reasons caution should be exercised when generalizing from the current findings. First, the national samples in this study were convenience samples. Therefore, they might neither be fully representative of their countries nor their countries’ general population. Also, information on the numbers of students who were asked, but refused to participate was not collected and is thus not available. Random sampling techniques should be employed in future research to overcome possible problems of sampling bias. Second, the measurement of individualism with five items and collectivism with five items might be inadequate for sampling the entirety of these constructs’ components. To better understand this issue, future research may benefit from using measures of self-construal. Also, horizontal and vertical facets of individualism and collectivism were conflated, and the scales did not include any reverse-coded items. This could have introduced response bias. Further, acquiescence bias, which tends to be stronger as a function of collectivism, arguably could have led to the overestimation of associations with collectivism, and the underestimation of associations with individualism. Third, the INDCOL scale was not administered in the home language at all study sites. The English INDCOL was used in Jordan, Palestine, Saudi Arabia, and Tunisia. Note that Palestine and Tunisia, together with Turkey and the USA, had the highest scores on individualism. There is evidence indicating that language itself might function as a prime (15). Thus, high individualism scores observed in Palestinian and Tunisian samples might be due to such effects, rather than truly reflecting the cultural orientation. Fourth, the cross-sectional study design does not allow for causal interpretations. To infer causality, prospective and longitudinal research designs are needed.

## Conclusion

In this study, we tested whether individualistic and collectivistic values are related to self-reported suicidal behavior, attitudes and psychological distress in a 12-nation sample of young adults. Our findings confirm and extend some findings from previous research.

The answer to the question we posed in the title is that universally both high and low individualism may be associated with suicide attempts. With regards suicidal ideation the answer depends on the cultural background. According to the current findings, higher individualism appears to be protective against suicidal ideation in Muslim collectivistic cultures, but seems a risk factor for suicidal ideation among Asian collectivistic cultures; both high and low individualism appears a risk factor in individualistic countries. At the same time, higher collectivism appears to protect against suicidal ideation among individualistic cultures, but not among Asian and Muslim collectivistic cultures. It is possible that individualistic values have a personally liberating and protective effect in some Muslim countries, where social pressure to conform is high. In other countries, where there is a generally lower level of social cohesion and/or less pressure to conform (Western individualistic and Asian collectivistic countries), higher individualism might further drive people at risk into isolation. Higher collectivism might be protective, when coupled with social support. Further, the findings from this study yielded interesting results concerning the attitudes towards suicide and suicidal persons. Collectivism may promote the communication of psychological problems, whereas hiding suicidal behavior is positively associated with individualism. Acceptability of suicide is negatively associated with collectivism, while both individualism and collectivism at the mean level are positively associated with social acceptance of a suicidal peer and emotional involvement. Individualism and collectivism are associated with disapproval of suicidal disclosure in a complex way, and associations also differ dependent on national suicide rate.

In this research, we adopted the view that, in their modest dosages, individualistic and collectivistic values correspond to individuality and relatedness, which in turn correspond to the two universal social human needs. The results suggest that, indeed, only intermediate levels of individualism may be considered protective against suicide attempts. If gratified at an optimum level, both value orientations could benefit people in many ways. For instance, collectivistic values may foster sharing, helping, and reciprocity, which in turn increase social cohesion and social support. Individualistic values, on the other hand, may help people get to know themselves better and to determine individual goals, based on what they may think that will make them happier. In contrast, overly individualistic or collectivistic values may shatter such possible benefits of these two fundamental value orientations.

## Data Availability Statement

The datasets generated for this study are available on request to the corresponding authors.

## Ethics Statement

The studies involving human participants were reviewed and approved by relevant institutional review boards. Except for Austria, where such an approval formally was not necessary, according to the relevant legal requirements and regulations, approval was obtained at all study sites. The patients/participants provided their written informed consent to participate in this study.

## Author Contributions

ME designed the study, organized the database, contributed to the statistical analysis, and wrote the first draft of the manuscript. UT performed the statistical analysis and wrote sections of the manuscript. All authors contributed to subsequent manuscript revisions, and read and approved the submitted version.

## Funding

This study was supported by the Open Access Publishing Fund of the University of Vienna.

## Conflict of Interest

The authors declare that the research was conducted in the absence of any commercial or financial relationships that could be construed as a potential conflict of interest.

## References

[1] Bertolote JM Fleishmann A Leo DD Bolhari J Botega N Silva DD Suicide attempts, plans, and ideation in culturally diverse sites: the WHO SUPRE-MISS community survey. Psychol Med (2005) 35:1457–65. 10.1017/S0033291705005404 16164769

[2] Värnik P Suicide in the world. Int J Environ Res Public Health (2012) 9:760–71. 10.3390/ijerph9030760 PMC3367275 22690161

[3] Jeon HJ Park JH Shim EJ Permissive attitude toward suicide and future intent in individuals with and without depression: results from a nationwide survey in Korea. J Nerv Ment Dis (2013) 201:286–91. 10.1097/NMD.0b013e318288d2c7 23538973

[4] Lenzi M Colucci E Minas H Suicide, culture, and society from a cross-national perspective. Cross Cult Res (2012) 46:50–71. 10.1177/1069397111424036

[5] Stack S Kposowa AJ The association of suicide rates with individual-level suicide attitudes: a cross-national analysis. Soc Sci Q (2008) 89:39–59. 10.1111/j.1540-6237.2008.00520.x

[6] Hofstede G Hofstede GJ Minkov M Cultures and Organizations – Software of the Mind: Intercultural Cooperation and its Importance for Survival. New York: McGraw-Hill (2010). p. 576.

[7] Kagitcibasi C Berry JW Cross-cultural psychology: current research and trends. Ann Rev Psychol (1989) 40:493–531. 10.1146/annurev.ps.40.020189.002425

[8] Triandis HC Individualism and Collectivism. Boulder, CO: Westview Press (1995). p. 288.

[9] Oyserman D The lens of personhood: viewing the self and others in a multicultural society. J Pers Soc Psychol (1993) 65:993–1009. 10.1037/0022-3514.65.5.993

[10] Cross SE Hardin EE Gercek-Swing B The what, how, why, and where of self-construal. Pers Soc Psychol Rev (2011) 15:142–79. 10.1177/1088868310373752 20716643

[11] Kitayama S Uskul AK Culture, mind, and the brain: current evidence and future directions. Ann Rev Psychol (2011) 62:419–49. 10.1146/annurev-psych-120709-145357 21126182

[12] Kühnen U Oyserman D Thinking about the self, influences thinking in general: cognitive consequences of salient self-concept. J Exp Soc Psychol (2002) 38:492–9. 10.1016/S0022-1031(02)00011-2

[13] Matsumoto D Yoo SH Nakagawa S Culture, emotion regulation, and adjustment. J Pers Soc Psychol (2008) 94:925–37. 10.1037/0022-3514.94.6.925 18505309

[14] Oyserman D Coon HM Kemmelmeier M Rethinking individualism and collectivism: evaluation of theoretical assumptions and meta-analyses. Psychol Bull (2002) 128:3–72. 10.1037/0033-2909.128.1.3 11843547

[15] Oyserman D Lee SW Does culture influence what and how we think? Effects of priming individualism and collectivism. Psychol Bull (2008) 134:311–42. 10.1037/0033-2909.134.2.311 18298274

[16] Durkheim E Suicide (transl. J. A. Spaulding). New York: Free Press (1897). p. 434. 1951.

[17] Hansen L Pritchard C Consistency in suicide rates in twenty-two developed countries by gender over time 1874-78, 1974-76, and 1998-2000. Arch Suicide Res (2008) 12:251–62. 10.1080/13811110802101153 18576206

[18] Kral MJ Suicide and the internalization of culture: three questions. Transcult Psychiatry (1998) 35:221–33. 10.1177/136346159803500203

[19] Maharajh HD Abdool PS Cultural aspects of suicide. Sci World J (2005) 5:736–46. 10.1100/tsw.2005.88 PMC5936491 16155688

[20] Chu JP Goldblum P Floyd R Bongar B The cultural theory and model of suicide. Appl Prev Psychol (2010) 14:25–40. 10.1016/j.appsy.2011.11.001

[21] Hjelmeland H Cultural research in suicidology: challenges andopportunities. Suicidol Online (2010) 1:34–52.

[22] Eskin M The effects of individualistic-collectivistic value orientations on non-fatal suicidal behavior and attitudes in Turkish adolescents and young adults. Scand J Psychol (2013) 54:493–501. 10.1111/sjop.12072 24111627

[23] Bryerton W That “every man for himself” thing: the rationales of individualism among the urban poor. Sociol Inq (2016) 86:79–102. 10.1111/soin.12102

[24] Waterman AS Individualism and interdependence. Am Psychol (1981) 36:762–73. 10.1037/0003-066X.36.7.762

[25] Wexler LM Gone JP Culturally responsive suicide prevention in indigenous communities: Unexamined assumptions and new possibilities. Am J Public Health (2012) 102:800–6. 10.2105/AJPH.2011.300432 PMC3483901 22420786

[26] Wiesenhutter E Suicide as an element of human freedom? Praxis Psychother (1971) 16:194–205.

[27] Bruffaerts R Demyttenaere K Hwang I Chiu WT Sampson N Kessler RC Treatment of suicidal people around the world. Br J Psychiatry (2011) 199:64–70. 10.1192/bjp.bp.110.084129 21263012 PMC3167419

[28] Husky MM McGuire L Flynn L Chrostowski C Olfson M Correlates of help-seeking behavior among at-risk adolescents. Child Psychiatry Hum Dev (2009) 40:15–24. 10.1007/s10578-008-0107-8 18581231

[29] Clement S Schauman O Graham T Maggioni F Evans-Lacko S Bezborodovs N What is the impact of mental health-related stigma on help-seeking? A systematic review of quantitative and qualitative studies. Psychol Med (2015) 45:11–27. 10.1017/S0033291714000129 24569086

[30] Curtis C Youth perceptions of suicide and help-seeking: ‘they’d think I was weak or “mental”. J Youth Stud (2010) 13:699–715. 10.1080/13676261003801747

[31] Gulliver A Griffiths KM Christensen H Perceived barriers and facilitators to mental health help-seeking in young people: a systematic review. BMC Psychiatry (2010) 10:113. 10.1186/1471-244X-10-113 21192795 PMC3022639

[32] Adolescents’ attitudes toward suicide, and a suicidal peer: a comparison between Swedish and Turkish high school students. Scand J Psychol (1995) 36:201–7. 10.1111/j.1467-9450.1995.tb00979.x 7644900

[33] Eskin M Voracek M Stieger S Altinyazar V A cross-cultural investigation of suicidal behavior and attitudes in Austrian and Turkish medical students. Soc Psychiatry Psychiatr Epidemiol (2011) 46:813–23. 10.1007/s00127-010-0254-7 20563550

[34] Eskin M Palova E Krokavcova M Suicidal behavior and attitudes in Slovak and Turkish high school students: a cross-cultural investigation. Arch Suicide Res (2014) 18:58–73. 10.1080/13811118.2013.803448 24350593

[35] Colucci E Cultural meaning(s) of suicide: a cross-cultural study. In: Colucci E Lester D, editors. Suicide and Culture: Understanding the Context. Cambridge, MA: Hogrefe (2012). p. 93–196.

[36] Eckersley R Is modern Western culture a health hazard? Int J Epidemiol (2006) 35:252–8. 10.1093/ije/dyi235 16303804

[37] Veenhoven R Quality-of-life in individualistic society. Soc Ind Res (1999) 48:159–88. 10.1023/A:1006923418502

[38] Eckersley R Dear K Cultural correlates of youth suicide. Soc Sci Med (2002) 55:1891–904. 10.1016/S0277-9536(01)00319-7 12406459

[39] Lester D Nations’ rated individualism and suicide and homicide rates. Psychol Rep (2003) 92:426. 10.2466/pr0.2003.92.2.426 12785623

[40] Rudmin WF Ferrada-Noli M Skolbekken JA Questions of culture, age and gender in the epidemiology of suicide. Scand J Psychol (2003) 44:373–81. 10.1111/1467-9450.00357 12887559

[41] Leeuwen N Rodgers R Régner I Chabrol H The role of acculturation in suicidal ideation among second-generation immigrant adolescents in France. Transcult Psychiatry (2010) 47:812–32. 10.1177/1363461510382154 21088105

[42] Scott G Ciarrochi J Deane FP Disadvantages of being an individualist in an individualistic culture: idiocentrism, emotional competence, stress, and mental health. Aust Psychol (2004) 39:143–54. 10.1080/00050060410001701861

[43] Du H Li X Lin D Tam CC Hopelessness, individualism, collectivism, and substance use among young rural-to-urban migrants in China. Health Psychol Behav Med (2014) 2:211–20. 10.1080/21642850.2014.888656 PMC4346056 25750778

[44] Ahuvia AC Individualism/collectivism and cultures of happiness: a theoretical conjecture on the relationship between consumption, culture and subjective well-being at the national level. J Happiness Stud (2002) 3:23–36. 10.1023/A:1015682121103

[45] Fischer R Boer D What is more important for national well-being: money or autonomy? A meta-analysis of well-being, burnout, and anxiety across 63 societies. J Pers Soc Psychol (2011) 101:164–84. 10.1037/a0023663 21604894

[46] Triandis HC Cultural syndrome and subjective well-being. In: Diener E Suh EM, editors. Culture and Subjective Well-Being. Cambridge, MA: MIT Press (2000). p. 87–112.

[47] Caldwell-Harris CL Ayçiçegi A When personality and culture clash: the psychological distress of allocentrics in an individualist culture and idiocentrics in a collectivist culture. Transcult Psychiatry (2006) 43:331–61. 10.1177/1363461506066982 17090622

[48] Ogihara Y Uchida Y Does individualism bring happiness? Negative effects of individualism on interpersonal relationships and happiness. Front Psychol (2014) 5:135. 10.3389/fpsyg.2014.00135 24634663 PMC3942875

[49] Rhodes AE Boyle MH Bridge JA Sinyor M Links PS Tonmyr L Antecedents and sex/gender differences in youth suicidal behavior. World J Psychiatry (2014) 4:120–32. 10.5498/wjp.v4.i4.120 PMC4274584 25540727

[50] Canetto SS Lester D Gender, culture, and suicidal behavior. Transcult Psychiatry (1998) 35:163–90. 10.1177/136346159803500201

[51] Schrijvers DL Bollen J Sabbe BG The gender paradox in suicidal behavior and its impact on the suicidal process. J Affect Disord (2012) 138:19–26. 10.1016/j.jad.2011.03.050 21529962

[52] Callanan VJ Davis MS Gender differences in suicide methods. Soc Psychiatry Psychiatr Epidemiol (2012) 47:857–69. 10.1007/s00127-011-0393-5 21604180

[53] Denning DG Conwell Y King D Cox C Method choice, intent, and gender in completed suicide. Suicide Life Threat Behav (2000) 30:282–8. 10.1111/j.1943-278X.2000.tb00992.x 11079640

[54] Payne S Swami V Stanistreet DL The social construction of gender and its influence on suicide: a review of the literature. J Mens Health (2008) 5:23–35. 10.1016/j.jomh.2007.11.002

[55] Wong YJ Wang SY Klann EM The emperor with no clothes: A critique of collectivism and individualism. Arch Sci Psychol (2018) 6:251–60. 10.1037/arc0000059

[56] Freeman MA Bordia P Assessing alternative models of individualism and collectivism: a confirmatory factor analysis. Europ J Pers (2001) 15:105–21. 10.1002/per.398

[57] Li F Aksoy L Dimensionality of individualism–collectivism and measurement equivalence of Triandis and Gelfand’s scale. J Bus Psychol (2007) 21:313–29. 10.1007/s10869-006-9031-8

[58] Inglehart R Welzel C (2010). The WVS cultural map of the world. http://pagines.uab.cat/seangolden/sites/pagines.uab.cat.seangolden/files/World%20Values%20Surveys%20maps%2020110606.pdf, [Accessed September 26, 2014].

[59] Eskin M Sun JM Abuidhail J Yoshimasu K Kujan O Janghorbani M Suicidal behavior and psychological distress in university students: a 12-nation study. Arch Suicide Res (2016) 20:369–88. 10.1080/13811118.2015.1054055 26954847

[60] Eskin M Kujan O Voracek M Shaheen A Carta MG Sun JM Cross-national comparisons of attitudes towards suicide and suicidal persons in university students from 12 countries. Scand J Psychol (2016) 57:554–63. 10.1111/sjop.12318 27538761

[61] Eskin M Poyrazli S Janghorbani M Bakhshi S Carta MG Tran US The role of religion on suicidal behavior, attitudes and psychological distress in university students: a multinational study. Transcult Psychiatry (2019) 56:853–77. 10.1177/1363461518823933 30734653

[62] Wasti SA Erdil ES Measurement of individualism and collectivism: validation of the self-construal scale and INDCOL in Turkish. Yönetim Araştırmaları Dergisi (2007) 7:39–66.

[63] Singelis TM Triandis HC Bhawuk DP Gelfand MJ Horizontal and vertical dimensions of individualism and collectivism: a theoretical and measurement refinement. Cross Cult Res (1995) 29:240–75. 10.1177/106939719502900302

[64] Goldberg DP Williams P A User"s Guide to the General Health Questionnaire. Windsor: NFER-Nelson (1988). p. 129.

[65] Goldberg DP Gater R Sartorius N Ustun TB Piccinelli M Gureje O The validity of two versions of the GHQ in the WHO study of mental illness in general health care. Psychol Med (1997) 27:191–7. 10.1017/S0033291796004242 9122299

[66] Eskin M The effects of religious versus secular education on suicide ideation and suicidal attitudes in adolescents in Turkey. Soc Psychiatry Psychiatr Epidemiol (2004) 39:536–42. 10.1007/s00127-004-0769-x 15243691

[67] Samejima F Estimation of a latent ability using a response pattern of graded scores. Psychometrika Monogr (1969) 34(Suppl. 4):1–100. 10.1007/BF03372160

[68] Kim ES Yoon M Testing measurement invariance: a comparison of multiple-group categorical CFA and IRT. Struct Equat Modeling (2011) 18:212–28. 10.1080/10705511.2011.557337

[69] Sass DA Testing measurement invariance and comparing latent factor means within a confirmatory factor analysis framework. J Psychoeduc Assess (2011) 29:347–63. 10.1177/0734282911406661

[70] Hu LT Bentler PM Cutoff criteria for fit indexes in covariance structure analysis: conventional criteria versus new alternatives. Struct Equat Modeling (1999) 6:1–55. 10.1080/10705519909540118

[71] Schermelleh-Engel K Moosbrugger H Müller H Evaluating the fit of structural equation models: tests of significance and descriptive goodness-of-fit measures. Meth Psychol Res Online (2003) 8:23–74.

[72] Steiger JH A note on multiple sample extensions of the RMSEA fit index. Struct Equat Modeling (1998) 5:411–9. 10.1080/10705519809540115

[73] Kenny DA Kaniskan B McCoach DB The performance of RMSEA in models with small degrees of freedom. Soc Meth Res (2014) 44:486–507. 10.1177/0049124114543236

[74] Muthén BO du Toit SHC Spisic D Robust inference using weighted least squares and quadratic estimating equations in latent variable modeling with categorical and continuous outcomes. (1997). http://www.statmodel.com/download/Article_075.pdf.

[75] Snijders TAB Bosker RJ Multilevel analysis. Los Angeles, CA: Sage (2012). p. 368.

[76] World Health Organization (2014). http://apps.who.int/gho/data/node.main.MHSUICIDE?lang=en [Accessed September 26, 2014].

[77] Bellin E Reconsidering the robustness of authoritarianism in the Middle East: lessons from the Arab Spring. Comp Polit (2012) 44:127–49. 10.5129/001041512798838021

[78] Göle N Gezi: anatomy of a public square movement. Insight Turkey (2013) 15:7–14.

[79] Ellis TE Rutherford B Cognition and suicide: two decades of progress. Int J Cognit Ther (2008) 1:47–68. 10.1521/ijct.2008.1.1.47

[80] Bakan D The duality of Human Existence: Isolation and Communion in Western Man. Boston: Beacon Press (1966). p. 242.

[81] Brewer MB The social self: on being the same and different at the same time. Pers Soc Psychol Bull (1991) 17:475–82. 10.1177/0146167291175001

[82] Guisinger S Blatt SJ Individuality and relatedness: evolution of a fundamental dialectic. Am Psychol (1994) 49:104–11. 10.1037/0003-066X.49.2.104

[83] Dadašev S Skruibis P Gailienė D Latakienė J Grižas A Too strong? Barriers from getting support before a suicide attempt in Lithuania. Death Stud (2016) 40:507–12. 10.1080/07481187.2016.1184725 27260844

[84] Freedenthal S Stiffman AR “They might think I was crazy”: young American Indians’ reasons for not seeking help when suicidal. J Adolesc Res (2007) 22:58–77. 10.1177/0743558406295969

[85] Reynders A Kerkhof AJ Molenberghs G Van Audenhove C Help-seeking, stigma and attitudes of people with and without a suicidal past: a comparison between a low and a high suicide rate country. J Affect Disord (2015) 178:5–11. 10.1016/j.jad.2015.02.013 25770477

[86] Reynders A Kerkhof AJFM Molenberghs G Van Audenhove C Attitudes and stigma in relation to help-seeking intentions for psychological problems in low and high suicide rate regions. Soc Psychiatry Psychiatr Epidemiol (2014) 49:231–9. 10.1007/s00127-013-0745-4 23896893

[87] Crowder MK Kemmelmeier M Untreated depression predicts higher suicide rates in US honor cultures. J Cross Cult Psychol (2014) 45:1145–61. 10.1177/0022022114534915

[88] Šedivy NZ Podlogar T Kerr DC De Leo D Community social support as a protective factor against suicide: a gender-specific ecological study of 75 regions of 23 European countries. Health Place (2017) 48:40–6. 10.1016/j.healthplace.2017.09.004 28934635

[89] Nader IW Tran US Baranyai P Voracek M Investigating dimensionality of Eskin’s Attitudes toward Suicide Scale with Mokken scaling and confirmatory factor analysis. Arch Suicide Res (2012) 16:226–37. 10.1080/13811118.2012.695271 22852784

