# Supplementary material for: Is Individualism Suicidogenic? Findings From a Multinational Study of Young Adults From 12 Countries
Source: Front Psychiatry. 2020 Apr 3;11:259. doi: 10.3389/fpsyt.2020.00259 (PMC7145967; doi:10.3389/fpsyt.2020.00259)
Supplement: Supplementary file 1 [file DataSheet_1.docx]

Supplementary Material

**Is individualism suicidogenic? Findings from a multi-national study of young adults from 12 countries**

**Table S1.** Tests of measurement invariance.

| Scale or subscale | χ^2^(*df*) | CFI | TLI | SRMR |
| --- | --- | --- | --- | --- |
| Individualism |  |  |  |  |
| Configural invariance | 1186.37(55)*** | .907 | .813 | 0.063 |
| **Partial invariance** | **1805.58(185)***** | **.866** | **.921** | **0.071** |
| Full invariance | 4802.81(285)*** | .628 | .856 | 0.098 |
| Collectivism |  |  |  |  |
| Configural invariance | 285.69(55)*** | .965 | .931 | 0.031 |
| **Partial invariance** | **626.01(145)***** | **.928** | **.945** | **0.038** |
| Full invariance | 3298.45(285)*** | .547 | .825 | 0.068 |
| Acceptability of suicide^a^ |  |  |  |  |
| Configural invariance | 3496.94(200)*** | .975 | .965 | 0.051 |
| **Full invariance** | **8718.13(542)***** | **.938** | **.968** | **0.088** |
| Punishment after death^a^ |  |  |  |  |
| Configural invariance | 560.96(50)*** | .997 | .994 | 0.023 |
| **Full invariance** | **2981.89(257)***** | **.983** | **.993** | **0.071** |
| Suicide as a sign of mental illness | | |  |  |
| Configural invariance^b^ | 444.42(21)*** | .998 | .997 | 0.018 |
| **Full invariance** | **1193.86(130)***** | **.995** | **.999** | **0.044** |
| Communicating psychological problems^a^ | |  |  |  |
| Configural invariance | 790.08(20)*** | .980 | .941 | 0.037 |
| **Full invariance** | **2803.46(182)***** | **.933** | **.978** | **0.071** |
| Hiding suicidal behavior |  |  |  |  |
| Configural invariance^b^ | 141.37(10)*** | .994 | .993 | 0.022 |
| **Full invariance** | **624.46(69)***** | **.973** | **.996** | **0.049** |
| Open reporting and discussion of suicide | |  |  |  |
| Configural invariance^b^ | 373.96(10)*** | .914 | .906 | 0.061 |
| **Full invariance** | **849.57(69)***** | **.817** | **.971** | **0.051** |
| Social acceptance |  |  |  |  |
| Configural invariance | 4211.36(10)*** | .965 | .942 | 0.056 |
| **Full invariance** | **5683.62(379)***** | **.955** | **.980** | **0.082** |
| Helping a suicidal friend^a^ |  |  |  |  |
| Configural invariance | 1933.39(90)*** | .968 | .946 | 0.054 |
| **Partial invariance** | **3994.57(307)***** | **.936** | **.969** | **0.080** |
| Full invariance | 6313.10(342)*** | .896 | .954 | 0.099 |
| Disapproval of suicidal disclosure^c^ | |  |  |  |
| Configural invariance | 1453.61(23)*** | .913 | .751 | 0.067 |
| **Full invariance** | **2651.64(202)***** | **.851** | **.951** | **0.076** |
| Emotional involvement |  |  |  |  |
| Configural invariance^b^ | 250.99(21)*** | .952 | .925 | 0.033 |
| **Full invariance** | **1313.63(130)***** | **.754** | **.938** | **0.056** |

*Note*. Final models are printed boldface. For all analyses, data from the USA and the UK were merged. ^a^ Data from China and Japan were merged for these analyses as well. ^b^ In order to get a testable over-identified model, loadings of items 1 and 2 of this scale were constrained to equality (i.e., equal loadings). ^c^ Item 1 of this scale was removed to obtain configural invariance.

**Table S2.** Means and standard deviations of the E-ATSS and E-SRSPS subscale scores per county.

|  | Attitudes towards suicide factors (E-ATSS) | | | | | | Reactions to suicidality factors (E-SRSPS) | | | |
| --- | --- | --- | --- | --- | --- | --- | --- | --- | --- | --- |
| Country | Acceptability of suicide | Punishment after death | Suicide as a sign of mental illness | Comm. psychol. problems | Hiding suicidal behavior | Open reporting and disc. of suic. | Social acceptance | Helping a suicidal friend | Disappr. of suicidal disclosure | Emotional involvement |
| Austria | 2.57 (0.96) | 1.81 (0.82) | 2.98 (1.02) | 3.89 (0.72) | 2.04 (0.91) | 3.46 (0.83) | 3.89 (0.62) | 4.31 (0.55) | 2.22 (0.68) | 2.70 (0.86) |
| China | 2.02 (0.76) | 2.10 (0.80) | 2.46 (0.96) | 3.53 (0.89) | 2.62 (0.96) | 2.12 (0.84) | 3.90 (0.65) | 3.95 (0.60) | 2.80 (0.72) | 3.53 (0.73) |
| Iran | 1.55 (0.73) | 4.00 (0.98) | 3.62 (1.20) | 3.59 (0.90) | 2.99 (1.07) | 2.97 (1.06) | 3.94 (0.76) | 4.16 (0.65) | 2.68 (0.74) | 3.38 (0.83) |
| Italy | 2.07 (0.84) | 1.92 (0.88) | 2.36 (1.10) | 4.01 (0.67) | 1.88 (0.99) | 3.54 (0.88) | 4.03 (0.60) | 4.27 (0.53) | 2.53 (0.69) | 3.20 (0.90) |
| Japan | 2.21 (0.84) | 2.19 (0.72) | 2.70 (0.96) | 3.57 (0.61) | 3.17 (0.76) | 2.82 (0.75) | 3.33 (0.67) | 3.84 (0.60) | 3.09 (0.65) | 2.71 (0.75) |
| Jordan | 1.88 (0.85) | 4.02 (0.94) | 3.93 (1.11) | 3.93 (0.89) | 3.13 (1.12) | 3.50 (1.11) | 3.66 (0.78) | 4.02 (0.81) | 3.18 (0.74) | 3.62 (0.85) |
| Palestine | 1.61 (0.81) | 3.68 (0.91) | 3.34 (1.18) | 3.64 (0.82) | 2.74 (1.06) | 3.10 (1.01) | 3.66 (0.88) | 3.92 (0.82) | 3.00 (0.74) | 3.48 (0.84) |
| Saudi Arabia | 2.08 (1.14) | 3.55 (1.11) | 3.30 (1.33) | 3.74 (1.14) | 2.95 (1.20) | 3.05 (1.08) | 3.54 (1.13) | 3.69 (1.17) | 3.13 (1.03) | 3.47 (1.07) |
| Tunisia | 1.41 (0.67) | 3.86 (0.83) | 3.47 (1.18) | 3.75 (0.78) | 2.90 (1.05) | 3.06 (0.92) | 4.01 (0.67) | 4.21 (0.63) | 2.62 (0.71) | 3.16 (0.81) |
| Turkey | 1.47 (0.67) | 3.67 (1.06) | 2.62 (1.20) | 3.84 (0.83) | 2.59 (1.08) | 2.82 (1.08) | 4.25 (0.62) | 4.29 (0.59) | 2.36 (0.80) | 3.78 (0.91) |
| UK | 2.50 (0.92) | 1.86 (0.88) | 2.66 (1.11) | 3.94 (0.79) | 1.92 (0.88) | 3.57 (0.82) | 3.97 (0.61) | 3.97 (0.53) | 2.22 (0.69) | 2.36 (0.89) |
| USA | 1.81 (0.75) | 2.67 (0.90) | 2.68 (1.06) | 4.08 (0.67) | 2.34 (0.92) | 2.90 (0.90) | 4.10 (0.64) | 4.03 (0.52) | 2.60 (0.70) | 3.03 (0.85) |

*Note*. Comm. psychol. problems = Communicating psychological problems; Open reporting and disc. of suic. = Open reporting and discussion of suicide; Disappr. of suicidal disclosure = Disapproval of suicidal disclosure.
